# Supplementary material for: High Expression of NRF2 Is Associated with Increased Tumor-Infiltrating Lymphocytes and Cancer Immunity in ER-Positive/HER2-Negative Breast Cancer
Source: Cancers (Basel). 2020 Dec 21;12(12):3856. doi: 10.3390/cancers12123856 (PMC7766649; doi:10.3390/cancers12123856)
Supplement: Supplementary file 1 [file cancers-12-03856-s001.pdf]

Supplemental Materials: High Expression of NRF2 Is Associated with Increased Tumor-Infiltrating Lymphocytes and Cancer Immunity in ER-Positive/HER2-Negative Breast Cancer

Masanori Oshi, Fernando A. Angarita, Yoshihisa Tokumaru, Li Yan, Ryusei Matsuyama, Itaru Endo and Kazuaki Takabe

Table S1. Correlation of the NRF2 expression with gene expressions of Hallmark_apoptosis gene set in ER-positive/HER2-negative breast cancer in the METABRIC and GSE96058 cohorts. Spearman's rank correlation coefficient was used to the analysis.

	METABRIC		GSE96058			METABRIC		GSE96058	
Genes	*r*	*p*	*r*	*p*	Genes	*r*	*p*	*r*	*p*
ADD1	-0.401	<0.01	0.24	<0.01	GPX1	0.254	<0.01	-0.025	0.22
AIFM3	0.143	<0.01	-0.113	<0.01	GPX3	0.068	0.01	0.293	<0.01
ANKH	0.184	<0.01	0.116	<0.01	GPX4	-0.13	<0.01	-0.102	<0.01
ANXA1	0.556	<0.01	0.329	<0.01	GSN	0.297	<0.01	0.373	<0.01
APP	0.268	<0.01	0.087	<0.01	GSR	-0.034	0.21	0.061	<0.01
ATF3	0.311	<0.01	0.265	<0.01	GSTM1	0.01	0.72	0.034	0.1
AVPR1A	0.044	0.1	0.227	<0.01	GUCY2D	-0.043	0.12	0.013	0.51
BAX	-0.076	<0.01	-0.188	<0.01	H1F0	-0.134	<0.01	-0.056	<0.01
BCAP31	-0.288	<0.01	-0.225	<0.01	HGF	0.114	<0.01	0.286	<0.01
BCL10	0.508	<0.01	0.282	<0.01	HMGB2	0.212	<0.01	-0.034	0.1
BCL2L1	-0.113	<0.01	-0.134	<0.01	HMOX1	-0.016	0.57	0.004	0.83
BCL2L10	0.041	0.13	0.138	<0.01	HSPB1	-0.174	<0.01	-0.151	<0.01
BCL2L11	0.145	<0.01	0.278	<0.01	IER3	0.009	0.74	0.043	0.04
BCL2L2	0.181	<0.01	0.268	<0.01	IFITM3	0.174	<0.01	0.109	<0.01
BGN	-0.104	<0.01	0	0.98	IFNB1	0.042	0.12	-0.044	0.03
BID	0.1	<0.01	0.057	<0.01	IFNGR1	-	-	0.408	<0.01
BIK	-0.124	<0.01	-0.091	<0.01	IGF2R	-0.177	<0.01	0.129	<0.01
BIRC3	0.126	<0.01	0.246	<0.01	IGFBP6	0.242	<0.01	0.257	<0.01
BMF	-0.184	<0.01	0.155	<0.01	IL18	-	-	0.11	<0.01
BMP2	0.005	0.85	0.263	<0.01	IL1A	0.135	<0.01	0.072	<0.01
BNIP3L	0.296	<0.01	0.186	<0.01	IL1B	0.319	<0.01	0.173	<0.01
BRCA1	-0.219	<0.01	-0.088	<0.01	IL6	0.3	<0.01	0.309	<0.01
BTG2	0.2	<0.01	0.246	<0.01	IRF1	-0.005	0.87	0.125	<0.01
BTG3	0.36	<0.01	0.18	<0.01	ISG20	0.019	0.48	0.003	0.89
CASP1	0.319	<0.01	0.265	<0.01	JUN	0.209	<0.01	0.26	<0.01
CASP2	0.194	<0.01	0.115	<0.01	KRT18	-0.117	<0.01	-0.149	<0.01
CASP3	0.039	0.15	0.088	<0.01	LEF1	0.149	<0.01	0.17	<0.01
CASP4	0.425	<0.01	0.32	<0.01	LGALS3	0.37	<0.01	0.21	<0.01
CASP6	0.396	<0.01	0.103	<0.01	LMNA	-0.306	<0.01	0.152	<0.01
CASP7	0.345	<0.01	0.174	<0.01	LUM	0.472	<0.01	0.078	<0.01
CASP8	0.416	<0.01	0.284	<0.01	MADD	-0.545	<0.01	0.085	<0.01
CASP9	0.115	<0.01	0.139	<0.01	MCL1	0.471	<0.01	0.273	<0.01
CAV1	0.449	<0.01	0.329	<0.01	MGMT	-0.018	0.51	-0.034	0.09
CCNA1	0.07	<0.01	0.208	<0.01	MMP2	0.13	<0.01	0.095	<0.01
CCND1	-0.301	<0.01	-0.045	0.03	NEDD9	0.314	<0.01	0.226	<0.01
CCND2	0.323	<0.01	0.231	<0.01	NEFH	0.05	0.07	0.107	<0.01
CD14	0.278	<0.01	0.147	<0.01	PAK1	-0.092	<0.01	0.02	0.32
CD2	-	-	0.15	<0.01	PDCD4	0.153	<0.01	0.209	<0.01
CD2	0.198	<0.01	.	.	PDGFRB	0.104	<0.01	0.139	<0.01
CD38	0.241	<0.01	0.158	<0.01	PEA15	-0.022	0.42	0.077	<0.01
CD44	-	-	0.095	<0.01	PLAT	0.026	0.33	0.038	0.06
CD44	0.033	0.22	.	.	PLCB2	-0.137	<0.01	0.121	<0.01
CD69	0.364	<0.01	0.244	<0.01	PMAIP1	0.115	<0.01	0.019	0.35
CDC25B	-0.241	<0.01	-0.028	0.16	PPP2R5B	-0.278	<0.01	0.026	0.19
CDK2	-0.03	0.27	0.016	0.42	PPP3R1	0.08	<0.01	0.24	<0.01
CDKN1A	0.161	<0.01	0.079	<0.01	PPT1	0.267	<0.01	0.129	<0.01

CDKN1B	0.416	<0.01	0.104	<0.01	PRF1	0.17	<0.01	0.185	<0.01
CFLAR	-	-	0.384	<0.01	PSEN1	0.37	<0.01	0.056	<0.01
CLU	0.164	<0.01	0.054	<0.01	PSEN2	-0.296	<0.01	-0.113	<0.01
CREBBP	0.153	<0.01	0.212	<0.01	PTK2	-0.316	<0.01	0.01	0.63
CTH	0.306	<0.01	0.119	<0.01	RARA	-0.338	<0.01	-0.081	<0.01
CTNNB1	0.388	<0.01	0.198	<0.01	RELA	0.132	<0.01	0.095	<0.01
CYLD	0.012	0.65	0.131	<0.01	RETSAT	-0.03	0.27	0.065	<0.01
DAP	-0.048	0.08	-0.039	0.05	RHOB	0.321	<0.01	0.092	<0.01
DAP3	0.04	0.15	-0.083	<0.01	RHOT2	-0.326	<0.01	-0.142	<0.01
DCN	0.471	<0.01	0.229	<0.01	RNASEL	-	-	0.165	<0.01
DDIT3	0.029	0.28	0.071	<0.01	ROCK1	0.536	<0.01	0.313	<0.01
DFFA	-0.038	0.17	0.223	<0.01	SAT1	0.285	<0.01	0.154	<0.01
DIABLO	0.056	0.04	-0.059	<0.01	SATB1	0.082	<0.01	0.262	<0.01
DNAJA1	0.27	<0.01	0.04	0.05	SC5D	-	-	0.133	<0.01
DNAJC3	-0.023	0.39	0.129	<0.01	SC5DL	0.121	<0.01	.	.
DNM1L	0.346	<0.01	0.056	<0.01	SLC20A1	-0.039	0.15	0.169	<0.01
DPYD	0.635	<0.01	0.393	<0.01	SMAD7	0.255	<0.01	0.084	<0.01
EBP	-0.112	<0.01	-0.254	<0.01	SOD1	0.136	<0.01	-0.119	<0.01
EGR3	0.265	<0.01	0.25	<0.01	SOD2	0.265	<0.01	0.257	<0.01
EMP1	0.365	<0.01	0.269	<0.01	SPTAN1	-0.165	<0.01	0.114	<0.01
ENO2	-0.247	<0.01	-0.131	<0.01	SQSTM1	-0.244	<0.01	-0.038	0.06
ERBB2	-0.296	<0.01	0.041	0.04	TAP1	-0.064	0.02	-0.002	0.94
ERBB3	-0.258	<0.01	-0.041	0.05	TGFB2	0.305	<0.01	0.164	<0.01
EREG	-	-	0.156	<0.01	TGFBR3	0.139	<0.01	0.322	<0.01
ETF1	0.427	<0.01	0.112	<0.01	TIMP1	-0.107	<0.01	0.113	<0.01
F2	-	-	-0.04	0.05	TIMP2	0.231	<0.01	0.105	<0.01
F2R	-	-	0.293	<0.01	TIMP3	0.195	<0.01	0.064	<0.01
FAS	0.477	<0.01	0.28	<0.01	TNF	-0.079	<0.01	0.119	<0.01
FASLG	0.087	<0.01	0.142	<0.01	TNFRSF12A	0.244	<0.01	0.059	<0.01
FDXR	0.095	<0.01	-0.175	<0.01	TNFSF10	0.165	<0.01	0.22	<0.01
FEZ1	0.209	<0.01	0.194	<0.01	TOP2A	-0.135	<0.01	-0.162	<0.01
GADD45A	0.321	<0.01	0.24	<0.01	TSPO	0.304	<0.01	-0.109	<0.01
GADD45B	0.149	<0.01	0.092	<0.01	TXNIP	0.295	<0.01	0.292	<0.01
GCH1	0.105	<0.01	-0.092	<0.01	VDAC2	0.252	<0.01	-0.055	<0.01
GNA15	-0.093	<0.01	0.074	<0.01	WEE1	0.327	<0.01	0.176	<0.01
					XIAP	-	-	0.091	<0.01

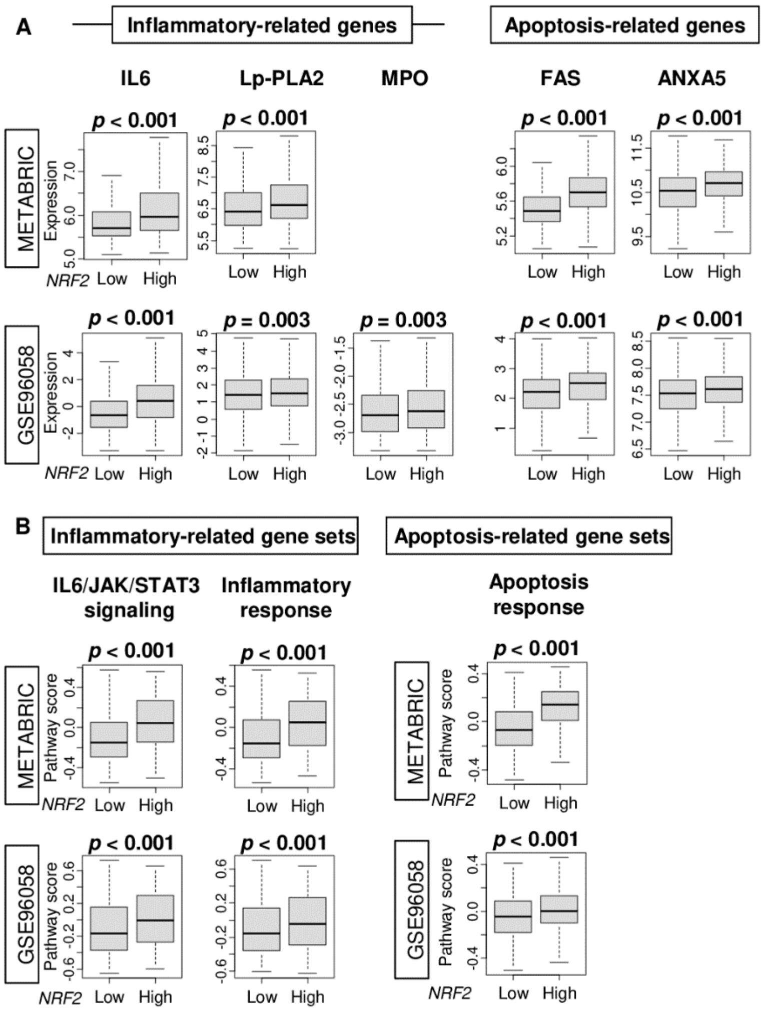

Figure S1. Association of the NRF2 with gene expression and pathway score of inflammatory- and apoptosis-related markers in ER+/HER2- breast cancer in the METABRIC and GSE96058 cohorts. Boxplots comparing (A) inflammatory-related genes (IL6, PLA2G7, and MPO) and apoptosis-related genes (FAS and ANXA5), and (B) inflammatory-related gene sets (IL6/JAK/STAT3 signaling), and inflammatory response pathways, and apoptosis pathway gene setas calculated by the GSVA algorithm, by low and high NRF2 groups. The cut-off of top tertile of NRF2 expression was considered as NRF2 high and low within each cohort. One-way ANOVA test was used for comparison. METABRIC cohort do not have MPO expression data.

Publisher's Note: MDPI stays neutral with regard to jurisdictional claims in published maps and institutional affiliations.

© 2020 by the authors. Licensee MDPI, Basel, Switzerland. This article is an open access article distributed under the terms and conditions of the Creative Commons Attribution (CC BY) license (http://creativecommons.org/licenses/by/4.0/).
